# New Oxaliplatin-Pyrophosphato Analogs with Improved In Vitro Cytotoxicity

**DOI:** 10.3390/molecules26113417

**Published:** 2021-06-04

**Authors:** Alessandra Barbanente, Rosa Maria Iacobazzi, Amalia Azzariti, James D. Hoeschele, Nunzio Denora, Paride Papadia, Concetta Pacifico, Giovanni Natile, Nicola Margiotta

**Affiliations:** 1 Dipartimento di Chimica, Università degli Studi di Bari Aldo Moro, Via E. Orabona 4, 70125 Bari, Italy; alessandra.barbanente@uniba.it (A.B.); concetta.pacifico@uniba.it (C.P.); giovanni.natile@uniba.it (G.N.); 2 Laboratorio di Farmacologia Sperimentale, IRCCS Istituto Tumori “Giovanni Paolo II”, O. Flacco St., 70124 Bari, Italy; rosamaria.iacobazzi@gmail.com (R.M.I.); a.azzariti@oncologico.bari.it (A.A.); 3 Department of Chemistry, Eastern Michigan University, Ypsilanti, MI 48197, USA; hoeschel@msu.edu; 4 Dipartimento di Farmacia-Scienze del Farmaco, Università degli Studi di Bari Aldo Moro, Via E. Orabona 4, 70125 Bari, Italy; nunzio.denora@uniba.it; 5 Department of Biological and Environmental Sciences and Technologies (DiSTeBA), University of Salento, Prov.le Lecce-Monteroni, Centro Ecotekne, 73100 Lecce, Italy; paride.papadia@unisalento.it

**Keywords:** cisplatin, oxaliplatin, phosphaplatins, pyrophosphate, bone tumors, antitumor drugs

## Abstract

Two new Pt(II)-pyrophosphato complexes containing the carrier ligands *cis*-1,3-diaminocyclohexane (*cis*-1,3-DACH) and *trans*-1,2-diamine-4-cyclohexene (1,2-DACHEX), variants of the 1*R*,2*R*-diaminocyclohexane ligand present in the clinically used Pt-drug oxaliplatin, have been synthesized with the aim of developing new potential antitumor drugs with high bone tropism. The complexes are more stable at physiological pH than in acid conditions, with Na_2_[Pt(pyrophosphato)(*cis*-1,3-DACH)] (**1**) slightly more stable than [Pt(dihydrogenpyrophosphato)(1,2-DACHEX)] (**2**). The greater reactivity at acidic pH ensures a greater efficacy at the tumor site. Preliminary NMR studies indicate that **1** and **2** react slowly with 5’-GMP (used as a model of nucleic acids), releasing the pyrophosphate ligand and affording the bis 5’-GMP adduct. In vitro cytotoxicity assays performed against a panel of four human cancer cell lines have shown that both compounds are more active than oxaliplatin. Flow cytometry studies on HCT116 cells showed that the pyrophosphato compounds with the non-classical 1,3- and 1,4-diaminocyclohexane ligands (**1** and **4**) are the most capable to induce cells’ death by apoptosis and necrosis.

## 1. Introduction

Cisplatin is one of the most potent drugs used for the treatment of a variety of cancers, including ovarian, testicular, small-cell lung, and colorectal cancer [1,2,3,4]. Severe side effects, such as nephro-, neuro-, and oto-toxicity, limit its use [5,6]. In order to find more effective chemotherapeutics with reduced toxicity, many new platinum complexes have been synthesized and tested for their anticancer activity. However, only carboplatin and oxaliplatin have obtained the Food and Drug Administration (FDA) approval [7]. Despite the progress in antitumor therapy, the search for innovative pharmacological strategies that can limit, at least in part, some of the unsolved therapeutic problems, remains a very active field of research [8]. A strategy for reducing the systemic side effects of Pt-based drugs consists in the choice of carrier ligands that promote the specific accumulation of the drug in target organs or cells [9,10,11]. One remarkable example is represented by bisphosphonate ligands (BPs), that can deliver the therapeutic agent specifically to the bone tissue, and can be ascribed to the Drug Targeting and Delivery (DTD) approach, together with the several platinum(II) complexes with phosphonate ligands already investigated [12,13,14,15,16,17,18,19]. Another class of platinum compounds with bone tropism is represented by phosphaplatins, where the platinum ion is coordinated to two *cisN*-donors and to a single bidentate pyrophosphate ligand. Initially reported by Bose et al., these compounds not only possess excellent water solubility and stability under physiological conditions [20], but also proved to be highly cytotoxic against human tumors resistant to cisplatin and carboplatin. Among phosphaplatins, the compound [Pt(dihydrogenpyrophosphato)(1*R*,2*R*-DACH)] (**3**), although exhibiting reduced cellular accumulation with respect to cisplatin in cisplatin- and carboplatin-resistant human ovarian cancer cells, unlike cisplatin, was able to overexpress fas and fas-related transcription factors as well as some proapoptotic genes (such as Bak and Bax) in treated cells [20]. Thus, phosphaplatins appear to act by cytotoxic mechanisms different from those of cisplatin, and their cytotoxicity does not appear to be linked to the limited amount of drug entering into the nucleus of the cell and forming covalent adducts with DNA, thereby overcoming DNA-repair-based resistance [20,21,22,23]. Compound **3** (also named PT-112) is currently undergoing Phase I/II clinical trials (NCTU2266745, NCTU3409458) in patients with advanced solid tumors. Interestingly, **3** was also found to induce immunogenic cell death in mouse tumor models. In this context, we found worthy to investigate the corresponding derivative of kiteplatin with the pyrophosphate ligand, namely [Pt(dihydrogenpyrophosphato)(*cis*-1,4-DACH)] (**4**) [24]. Unlike phosphaplatins, compound **4** was found to form platinum adducts with DNA [25].

Apart from 1*R*,2*R*-DACH used in **3** and *cis*-1,4-DACH used in **4**, two more variants of cyclohexanediamine have been taken into consideration in past investigations: the *trans*-1,2-diamino-4-cyclohexene (1,2-DACHEX) and the *cis*-1,3-diaminocyclohexane (*cis*-1,3-DACH). In the case of 1,2-DACHEX, the introduction of a double bond in the cyclohexane ring greatly reduces its flexibility while increasing its planar extension. In addition, by chemical modifications at the double bond, it is possible to harbor targeting and/or dual-acting moieties. Four Pt(II) complexes with general formula [PtX_2_(1,2-DACHEX)] (X_2_ = Cl_2_, I_2_, 1,1′-cyclobutanedicarboxylate, and oxalate) were synthesized and tested against a panel of human tumor cell lines. They proved to have, in general, equal if not better cytotoxic activity than cisplatin and oxaliplatin and to be able to overcome the oxaliplatin resistance, thus showing how a limited structural variation at the diamine carrier ligand can deeply impact on the spectrum of activity of platinum drugs [26]. It is also worth mentioning that very recently, Hoeschele et al. have evaluated the role of the size of the alkane ring in complexes of general formula [PtCl_2_(*cis*-1,3-diaminocycloalkane)], where the cycloalkane is cyclobutane, cyclopentane, or cyclohexane. They have found that the derivative containing the cyclohexane ring, [PtCl_2_(*cis*-1,3-DACH)], kills cancer cells with greater efficacy than the other two derivatives containing the cyclopentane or the cyclobutane ring, and cisplatin [27].

**Figure 1:**
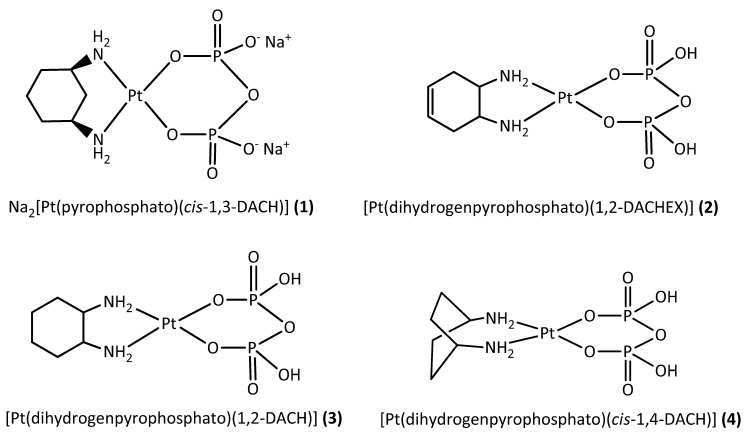
Pt(II)-pyrophosphato complexes.

Based on the above results, we have now extended the investigation to two new Pt(II)-pyrophosphato complexes (**1** and **2**; Figure 1) containing, respectively, the *cis*-1,3-diaminocyclohexane (*cis*-1,3-DACH) and the *trans*-1,2-diamino-4-cyclohexene (1,2-DACHEX) carrier ligands, which, together with compounds **3** and **4**, complete the set of possible variations at the cyclohexane ring [21,24]. In all cases, the presence of the pyrophosphate ligand ensures a high tropism toward bone tumors. The new complexes were synthesized and fully characterized, and, in the perspective of a potential in vivo application, their stability was investigated in a physiological-like buffer as well as in acidic conditions to simulate the low-pH environment typically surrounding a tumor mass. Moreover, in order to have a preliminary indication of the mechanism of action, we investigated the reactivity of **1** and **2** with 5’-GMP, used as the simplest model of DNA. Finally, the two Pt(II)-pyrophosphato complexes were tested to assess their in vitro cytotoxicity against a panel of human tumor cell lines.

## 2. Results and Discussion

### 2.1. Synthesis and NMR Characterization

The phosphaplatin derivative **3**, also known as PT-112, is currently under clinical trials for the treatment of advanced solid tumors, either alone or in combination with avelumab (a programmed cell death, PD-1 inhibitor). Interestingly, complex **3** is capable of inducing an immunogenic form of cancer cell death [28]. Inspired by these results and pursuing our interest on the pharmacological effect of small modifications in the diamine carrier ligand (which already led to the synthesis of compound **4** containing the *cis*-1,4-DACH ligand), we have now prepared two more phosphaplatin analogues of **3** bearing the *cis*-1,3-DACH (**1**) and the 1,2-DACHEX carrier ligands (**2**).

**Scheme 1:**
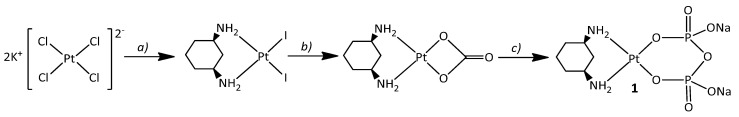
(a) Water, excess KI (8-fold), 5 min, room temperature, and then *cis*-1,3-diamino-cyclohexane (*cis*-1,3-DACH), 40 °C, 2.5 h. (b) Water, Ag_2_CO_3_, 40 °C, 2 h. (c) Water, pH = 8, sodium pyrophosphate decahydrate at 55 °C for 3 h, and then pH = 6 at room temperature for 30 min.

Na_2_[Pt(pyrophosphato)(*cis*-1,3-DACH)] (**1**) was prepared following a two-step reaction. The precursor complex [Pt(CO_3_)(*cis*-1,3-DACH)] was obtained by treating [PtI_2_(*cis*-1,3-DACH)] with Ag_2_CO_3_ in water. The carbonate precursor was then treated with a slight defect of sodium pyrophosphate at pH = 8.0, affording the desired compound **1**, which was obtained as a disodium salt by precipitation with acetone (Scheme 1).

**Figure 2:**
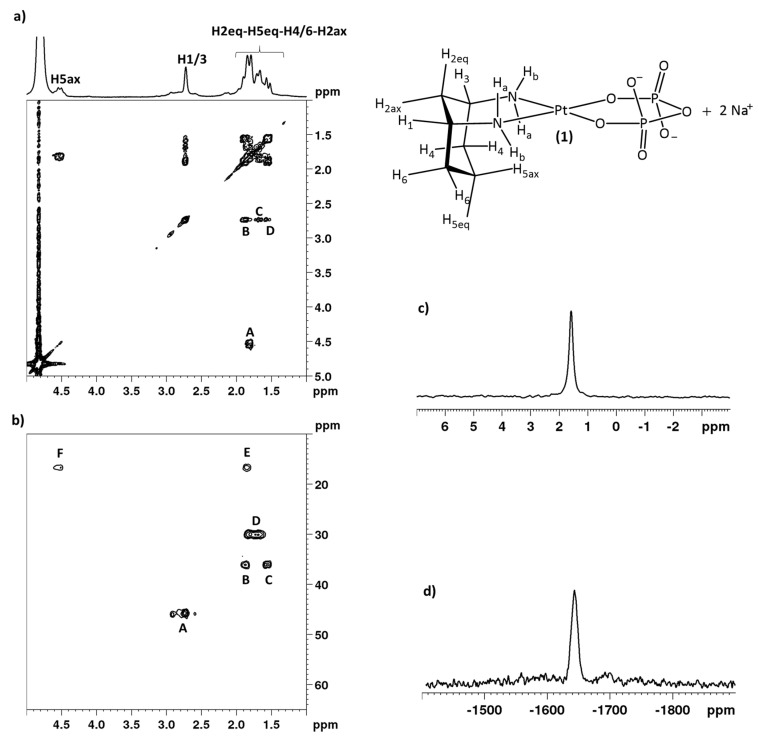
(**a**) COSY (300 MHz), (**b**) [^1^H-^13^C] HSQC (^13^C, 75.5 MHz), (**c**) ^31^P{^1^H} (121.5 MHz), and (**d**) ^195^Pt{^1^H} (64.5 MHz) NMR spectra of **1** dissolved in D_2_O.

Compound **1** was characterized by elemental analysis, ESI-MS, and multinuclear 1D and 2D NMR. The ESI-MS spectrum showed the presence of a peak at *m/z* = 551.9617, corresponding to [**1**+Na]^+^, with the experimental isotopic pattern of the peak in good agreement with the theoretical one. Both the elemental analysis and the ESI-MS are in agreement with the formation of a complex having Pt and pyrophosphate in a 1:1 ratio. The ^1^H-NMR characterization of compound **1** was carried on with the help of a COSY spectrum in D_2_O (Figure 2a). The cross-peak A correlates the signals at 4.53 and 1.85 ppm that were assigned to H_5ax_ and H_5eq_, respectively. Of note is the huge de-shielding of H_5ax_, which indicates that the cyclohexane ring is forced in a rigid chair conformation with H_5ax_ falling in the de-shielding cone of the Pt atom. The cross-peaks which correlate the signals at 2.73/1.88 (B) and at 2.73/1.56 (D) ppm were assigned to H_1/3_/H_2eq_ and H_1/3_/H_2ax_, respectively. Finally, the signals at 2.73 and 1.74 ppm, which are correlated by cross-peak C, could be assigned to the methynic protons H_1/3_ and H_4/6_, respectively. In the [^1^H,^13^C] HSQC spectrum (Figure 2b), the cross-peaks (^13^C/^1^H) falling at 16.74/1.85 (F) and 16.74/4.53 (E) ppm confirmed the huge separation (2.68 ppm) between the H_5_ axial and the H_5_ equatorial protons. The cross-peak observed at 30.00/1.74 (D) ppm allowed the assignment of C_4/6_, while the cross-peaks falling at 36.13/1.88 (B) and 36.13/1.56 (C) ppm (^13^C/^1^H) belong to C_2_/H_2eq_ and C_2_/H_2ax_, respectively. Finally, cross-peak A, resonating at 45.95/2.73 ppm, was assigned to C_1/3_. The ^31^P NMR of **1** dissolved in D_2_O is reported in Figure 2c. A single peak with unresolved platinum satellites (platinum satellites usually broaden with an increase in molecular size and chemical shift anisotropy) [29,30] and falling at 1.58 ppm was assigned to the two phosphorous atoms of coordinated pyrophosphate ligand. This chemical shift is downfield if compared to that of free pyrophosphate (−6.35 ppm) measured in the same experimental conditions. The presence of a single peak in the ^31^P NMR supports the formation of a symmetric complex with both phosphate groups coordinated to Pt through one deprotonated oxygen atom. The ^195^Pt NMR of **1** dissolved in D_2_O is reported in Figure 2d, and shows a single peak falling at –1643.31 ppm. This signal is in the range typical for a Pt(II) atom in an O_2_N_2_ coordination environment and is similar to those found in [Pt(dihydrogenpyrophosphato)(1*R*,2*R*-DACH)] [21,22] and [Pt(dihydrogenpyrophosphato)(*cis*-1,4-DACH)] [24].

Another interesting ligand that we have recently investigated is the *trans*-1,2-diamino-4-cyclohexene (1,2-DACHEX) [26,31]. With respect to 1*R*,2*R*-DACH, this ligand contains a double bond in the cyclohexane ring that greatly reduces its flexibility while extending its planarity. Similar to 1,2-DACH, 1,2-DACHEX can also be present in two asymmetric forms: 1*R*,2*R* and 1*S*,2*S*; however, due to the smaller puckering of 1,2-DACHEX with respect to the 1,2-DACH ring, we expect a smaller dependence of the overall stereochemistry of the complex upon the chirality of the diamine and used the racemic mixture. The 1,2-DACHEX analogue of oxaliplatin, [Pt(OXA)(1,2-DACHEX)] (OXA = oxalate), previously investigated, was able to overcome the oxaliplatin resistance in colorectal cancer cells and to induce the formation of lipid droplets in this cell line, thus suggesting a mechanism of action which includes severe metabolic stress. These results prompted us to prepare the 1,2-DACHEX analog of **3**, [Pt(dihydrogenpyrophosphato)(1,2-DACHEX)] (**2**). Compound **2** was obtained by a one-pot synthesis in water, starting from [Pt(CO_3_)(DMSO)_2_] (this latter was obtained by reacting [PtCl_2_(DMSO)_2_] with Ag_2_CO_3_). The carbonate precursor was treated, in water, with a slight defect of sodium pyrophosphate and then with 1 equivalent (0.121 mmol) of 1,2-DACHEX at pH = 8.0 (Supplementary Materials, Scheme S1). The precipitation of the desired product **2** was achieved by first lowering the pH of the mother solution to 1.0 with nitric acid and then adding acetone (the bis-protonated neutral form of **2** is insoluble in water/acetone).

**Figure 3:**
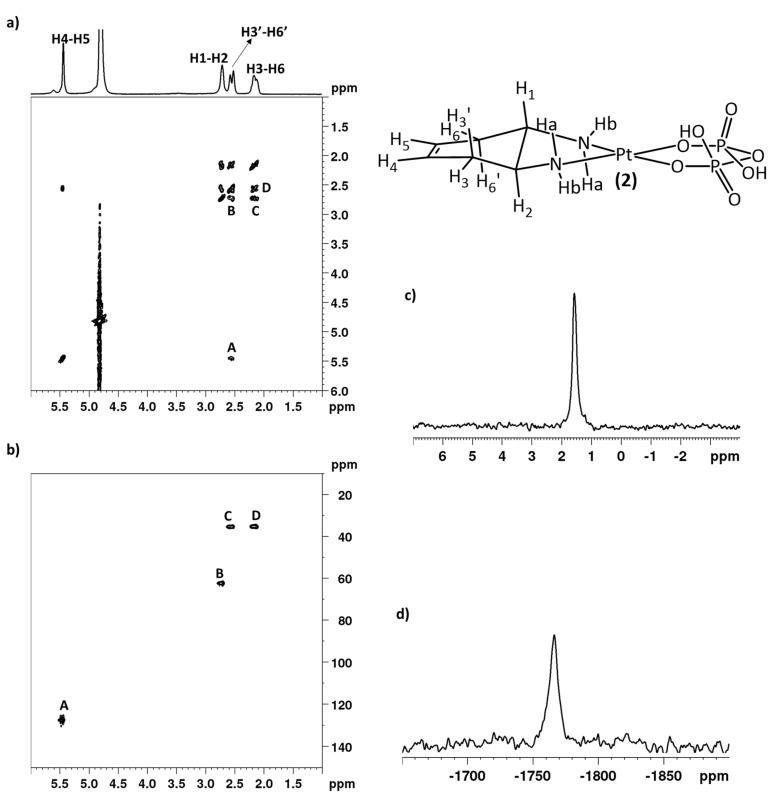
(**a**) COSY (300 MHz), (**b**) [^1^H-^13^C] HSQC (75.5 MHz, ^13^C), (**c**) ^31^P{^1^H} (121.5 MHz), and (**d**) ^195^Pt{^1^H} (64.5 MHz) NMR spectra of **2** in D_2_O (pH* = 9).

Compound **2** was characterized by elemental analysis, ESI-MS, and multinuclear 1D and 2D NMR. The ESI-MS spectrum showed the presence of a peak at *m/z* = 481.9797 corresponding to [**2**-H]^−^, and the experimental isotopic pattern of the peak was in good agreement with the theoretical one (data not shown). Both the elemental analysis and ESI-MS indicated the formation of a complex having Pt and pyrophosphate in a 1:1 ratio. The COSY spectrum of compound **2** in D_2_O (pH* = 10; pH* indicates pH reading from the pH-meter for D_2_O solutions, see also Material and Methods) is reported in Figure 3a. The cross-peak linking the signals at 5.45 and 2.56 ppm (A in Figure 3a) assigns these two resonances to the vinylic protons H_4/5_ and H_3*’*_, respectively. The cross-peaks at 2.72/2.56 and 2.72/2.16 ppm (B and C in Figure 3a, respectively) assign these resonances to H_1_/H_6*’*_ and H_1_/H_6_, respectively. Finally, the cross-peak which correlates the signals at 2.56 and 2.16 (D) ppm assigns these resonances to protons H_3*’*_ and H_3_, respectively.

In the [^1^H,^13^C] HSQC spectrum (Figure 3b), the cross-peaks (^13^C/^1^H) observed at 127.76/5.45 (A) and 62.43/2.72 (B) ppm were assigned to C_4/5_/H_4/5_ and C_1/2_/H_1/2_, respectively. Finally, the cross-peaks (^13^C/^1^H) falling at 35.43/2.56 (C) and 35.43/2.16 (D) ppm allowed the assignment of C_3/6_. The ^31^P NMR of **2** in D_2_O (Figure 3c) exhibits a single peak with unresolved platinum satellites falling at 1.79 ppm and assigned to the phosphorous atoms of the coordinated pyrophosphate ligand. Additionally, in this case, the presence of a single peak in the ^31^P NMR is evidence in support of the formation of a symmetric complex with both phosphate groups coordinated to Pt through one oxygen atom. The ^195^Pt NMR of **2** in D_2_O (pH* = 9) is reported in Figure 3d. A single peak falling at −1766 ppm is in agreement with a Pt(II) atom in a O_2_N_2_ coordination environment, as also found for complexes **1**, **3** [21,22], and **4** [24].

**Figure 4:**
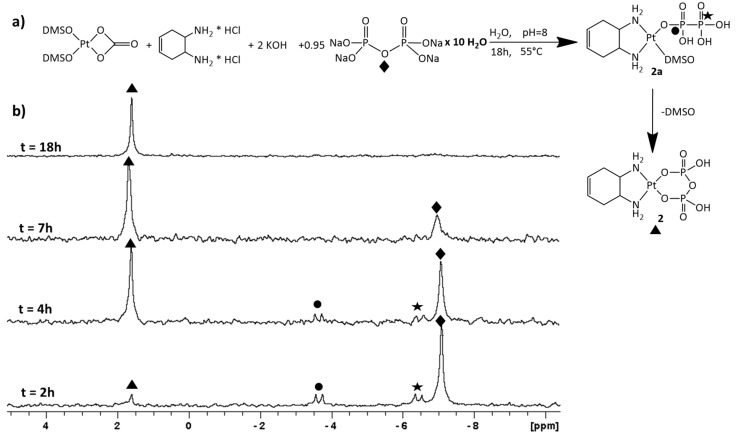
(**a**) Scheme of the reaction mechanism for the formation of complex **2**. (**b**) **^31^**P NMR spectra of the reaction mixture in H_2_O/D_2_O (90:10) recorded at different time intervals.

With the aim of investigating the mechanism of formation of complex **2**, we recorded the ^31^P NMR spectra of the reaction mixture in H_2_O/D_2_O (90:10) at different time intervals (Figure 4). The ^31^P spectrum recorded after 2 h of reaction showed, apart from the signal of free pyrophosphate (marked with a full diamond in Figure 4), two doublets falling at −6.42 and −3.65 ppm that were confidently assigned to the two phosphorous atoms of a mono-coordinated pyrophosphate in the intermediate complex **2a** (marked with a full star and a full circle in Figure 4). The coordination environment of this intermediate complex is completed by one DMSO ligand still bound to the Pt atom. After 2 h, we could also observe a small signal falling at 1.61 ppm (marked with a full triangle in Figure 3) belonging to complex **2**. The ^31^P NMR spectra recorded after 4 and 7 h revealed a decrease in intensity of the signal of the free pyrophosphate with the simultaneous increase of the signal at 1.61 ppm. The latter, after 18 h reaction, represents the only signal accounting for the total phosphorus and indicates the end of the reaction.

The synthetic procedure used for the synthesis of compound **2** could be applied, with some advantages, also to the synthesis of phosphaplatin **3**, [Pt(dihydrogenpyrophosphato)(1*R*,2*R*-DACH)], firstly reported by Bose et al. [21] (Supplementary Materials, Scheme S2). Differently from Bose, we could prepare complex **3** from the carbonate precursor [Pt(CO_3_)(1*R*,2*R*-DACH)], that can be easily prepared in water solution in high yield and is very reactive towards more strongly coordinating ligands such as pyrophosphate. Thus, by using the carbonate precursor, which, in addition, is more soluble in water than the [PtCl_2_(1*R*,2*R*-DACH)] precursor used in Bose’s procedure, we could reduce the reaction time and increase the yield.

### 2.2. NMR Experiments at Different pH Values and Calculation of pKa Values

**Figure 5:**
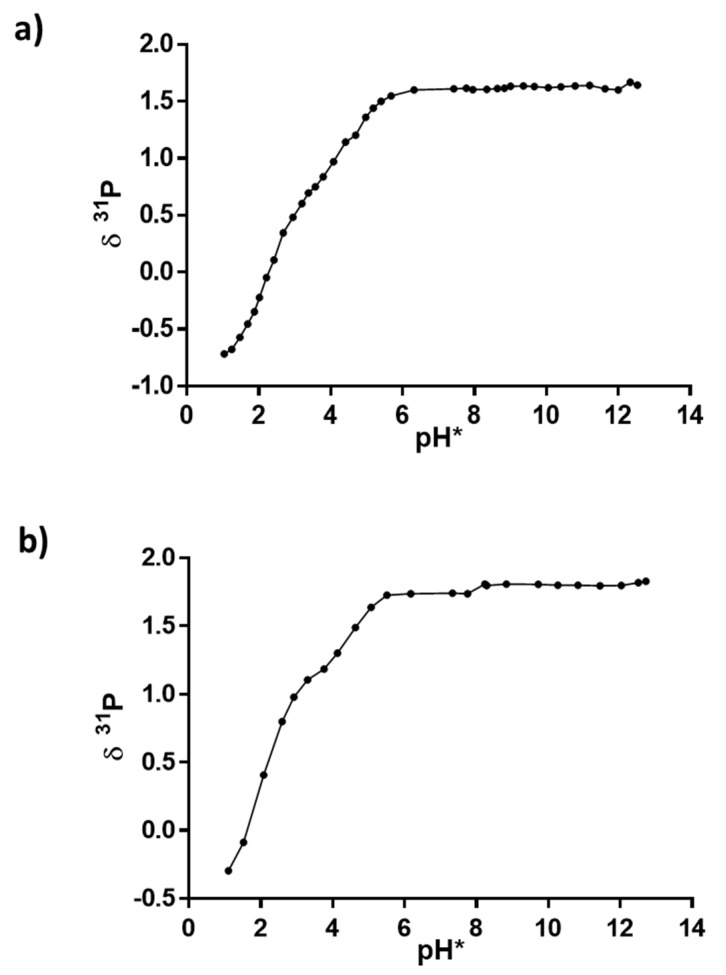
Plot of ^31^P chemical shift vs. pH* for **1** (**a**) and **2** (**b**).

**Scheme 2:**
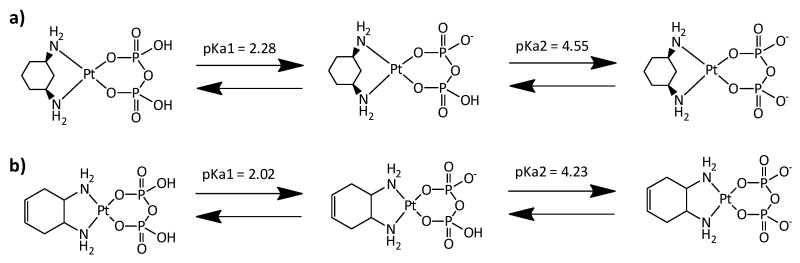
Protonation/deprotonation steps for **1** (**a**) and **2** (**b**) with corresponding pK_a1_ and pK_a2_.

Pyrophosphate, being a tetrabasic acid, has four ionization equilibria with pK_a_ values corresponding to pK_a1_ = 0.85, pK_a2_ = 1.49, pK_a3_ = 5.77, and pK_a4_ = 8.22 [32]. The pH titration of complexes **1** and **2** was carried on by recording ^31^P NMR spectra at different pH* values (Figure 5). The titration curves plotting the ^31^P chemical shift as a function of pH* exhibit changes in the slope (inflection points) corresponding to the various deprotonation steps (Scheme 2). As the pH* of the samples was increased from ca. 1.0 to 2.5, the ^31^P resonance shifts to lower field with an inflection point (calculated by fitting the points in this pH range to the Henderson–Hasselbach equation) falling at pH* = 2.28 and 2.02 for complexes **1** and **2**, respectively. Very likely, these pH* values correspond to the first deprotonation step of **1** and **2** (pK_a1_ in Scheme 2). By further increasing the pH from 2.5 to 6.0, we observed a further de-shielding of the phosphorous nuclei, with a second inflection point falling at pH* = 4.55 and 4.23 for compounds **1** and **2**, respectively. These pH values correspond to the second deprotonation of the coordinated pyrophosphate (pK_a2_ in Scheme 2). Further increase of pH* to ca. 13 had no significant effect on the ^31^P chemical shift. The pKa values obtained for **1** and **2** are comparable to those reported for **3** [21] and **4** [24], for which a similar biphasic profile, corresponding to two protonation/deprotonation steps, was observed (pK_a1_ = 2.06 and pK_a2_ = 4.40 for **3** and pK_a1_ = 2.03 and pK_a2_ = 4.47 for **4**).

### 2.3. Stability in Physiological Conditions

**Figure 6:**
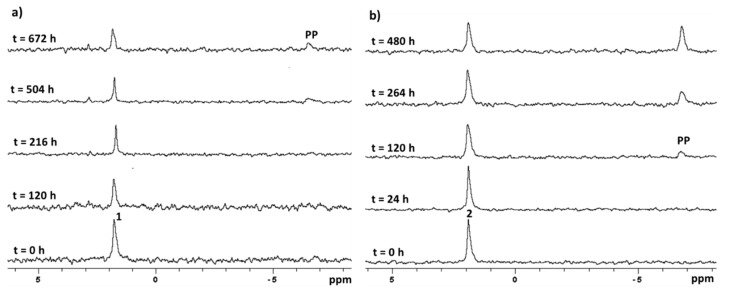
**^31^**P NMR (121.5 MHz) spectra at different times of **1** (**a**) and **2** (**b**) in physiological conditions (D_2_O, HEPES buffer 50 mM, pH* = 7.4, 120 mM NaCl, 37 °C).

To investigate the stability of **1** and **2** in physiological-like conditions, their solutions in D_2_O containing HEPES buffer (50 mM, pH = 7.4) and NaCl (120 mM) were monitored at 37 °C by ^31^P NMR spectroscopy. As can be seen from Figure 6a, at pH = 7.4 and 37 °C, compound **1** resulted to be very stable, and only after 672 h (28 days) could a small new signal falling at −6.54 ppm and assignable to free pyrophosphate be observed. This result is in line with the data reported in the literature for the analogous complexes **3** [21] and **4** [24], which also were quite stable but not to the extent of compound **1**. Compound **2** was significantly less stable than **1** and kept at pH = 7.4 and 37 °C, showed the appearance of a small ^31^P NMR signal falling at –6.54 ppm, and corresponding to free pyrophosphate, already after 120 h (Figure 6b). However, overall, also compound **2** can be considered stable for the time necessary to perform the in vitro cytotoxicity tests (72 h, see following section).

### 2.4. Stability in Acidic Environment

**Figure 7:**
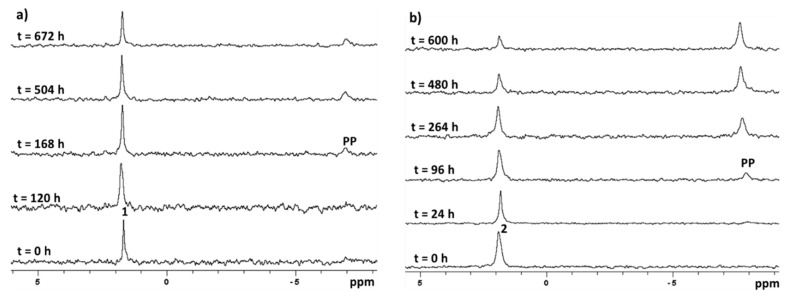
**^31^**P NMR (121.5 MHz) spectra at different times of **1** (**a**) and **2** (**b**) in acidic conditions (D_2_O, HEPES buffer 50 mM, pH* = 6.5, 120 mM NaCl, 37 °C).

To simulate the acidic environment surrounding cancer tissues, the stability of **1** and **2** was also investigated at pH* = 6.5 and 37 °C by ^31^P NMR spectroscopy. As can be seen from Figure 7a, compound **1** resulted to be still stable, and only after 168 h could a small ^31^P NMR signal falling at −6.91 ppm and corresponding to free pyrophosphate be observed. Similar to what was observed at pH* = 7.4, also at acid pH, complex **2** resulted to be slightly less stable than complex **1**, with the appearance of a small peak of free pyrophosphate already after 96 h (Figure 7b). The decrease in stability of both Pt-pyrophosphate complexes at acid pH suggests that they can release the pyrophosphate ligand, and form the pharmacologically active species, preferentially at the more acidic tumor site than in the blood stream or in the healthy tissues (a drug targeting and delivery advantage).

### 2.5. Reaction with 5’-GMP

**Figure 8:**
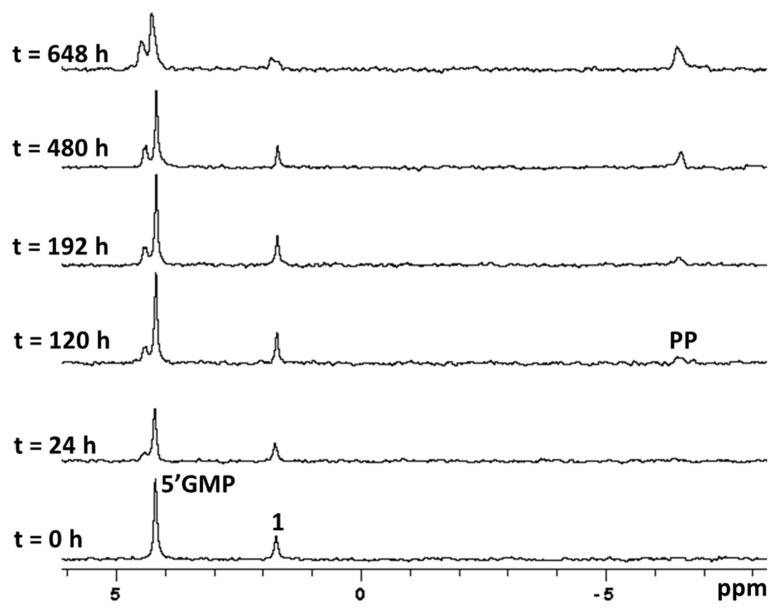
^31^P NMR spectra at different time intervals of **1** (5 mM) in the presence of 5’-GMP (12.5 mM) at pH* = 7.4 (D_2_O, 50 mM HEPES buffer, 120 mM NaCl, 37 °C).

**Figure 9:**
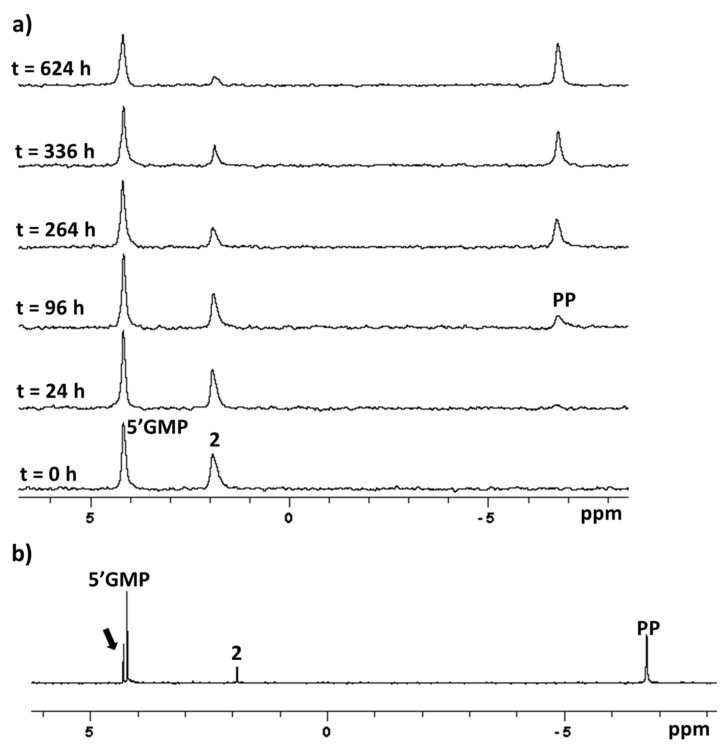
(**a**) ^31^P NMR spectra (121.5 MHz) at different time intervals of **2** (5 mM) in the presence of 5’-GMP (12.5 mM) at pH* = 7.4 (D_2_O, 50 mM HEPES buffer, 120 mM NaCl, 37 °C). (**b**) ^31^P NMR spectrum (202.3 MHz) of **2** (5 mM) in the presence of 5’-GMP (12.5 mM) at pH* = 7.4 (D_2_O, 50 mM HEPES buffer, 120 mM NaCl, 37 °C) recorded after 624 h.

A peculiar feature of phosphaplatins is that, unlike cisplatin, their pharmacological activity could not depend upon reaction with DNA [23]. However, in a previous work, the pyrophosphate derivative **4** was shown to be able to bind DNA [25], and therefore it could be worth also investigating the reaction of compounds **1** and **2** with DNA. As a preliminary step, we monitored by ^31^P and ^1^H NMR the reaction of **1** and **2** with 5*’*-GMP (2.5 equivalents, 0.010 mmol) in 50 mM HEPES buffer (pH* = 7.4) and 120 mM NaCl at 37 °C. The ^31^P spectrum, recorded soon after mixing of the reactants, showed only the peaks of free 5*’*-GMP (4.20 ppm) and of the starting substrates: 1.74 ppm for complex **1** (Figure 8) and 1.91 ppm for complex **2** (Figure 9a). In both cases, with time, it was possible to observe the appearance of a new peak at −6.49 ppm assigned to free pyrophosphate and a decrease in intensity of the signal belonging to the starting substrates. However, only in the case of compound **1** was a new peak at 4.43 ppm (Figure 8) clearly evident, which could be confidently assigned to the bis-adduct [Pt(5*’*-GMP)_2_(*cis*-1,3-DACH)].

**Figure 10:**
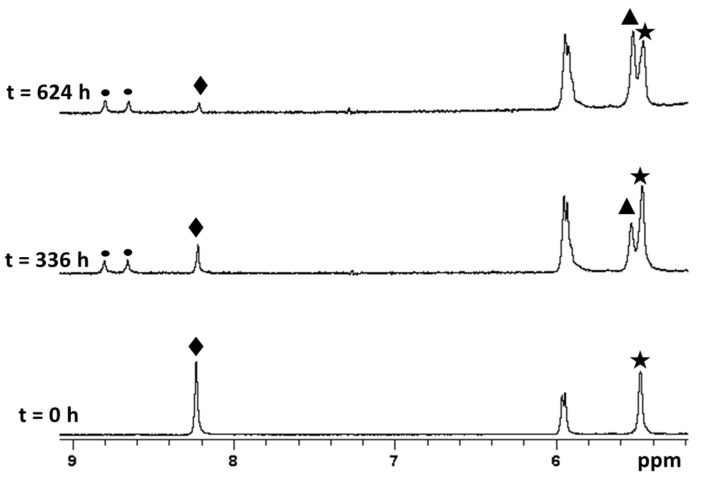
^1^H NMR spectra at different times of **2** (5 mM) in the presence of 5’-GMP (12.5 mM) at pH* = 7.4 (D_2_O, 50 mM HEPES buffer, 120 mM NaCl, 37 °C). Diamonds indicate free 5’-GMP, circles indicate coordinated 5’-GMP, stars indicate starting complex **2**, and triangles indicate the bis-adduct [Pt(5’-GMP)_2_(1,2-DACHEX)].

We argued that the absence of a separated signal downfield of that of free 5*’*-GMP (which could indicate the formation of the *bis*-adduct [Pt(5’-GMP)_2_(1,2-DACHEX)]) in the case of compound **2** could be due to the slight broadness of these signals, which could mask the separation between free and coordinated 5’-GMP (Figure 9a). Therefore, in the case of **2**, the ^31^P NMR spectrum was also recorded on a 500 MHz NMR instrument; as expected, a downfield peak falling at 4.27 ppm was observed (marked with an arrow in Figure 9b) that could be assigned to the coordinated 5’-GMP in the *bis*-adduct. The reaction of **2** with 5’-GMP was also monitored by ^1^H NMR spectroscopy (Figure 10). The spectrum recorded at time 0 showed the signals of the free nucleotide (e.g., the sharp singlet at 8.23 ppm belonging to proton H8 of guanine and marked with a full diamond in Figure 10) and the signals of the starting substrate (e.g., the vinylic protons of coordinated *trans*-1,2-diamine-4-cyclohexene marked with a full star in Figure 10). After 336 h, 3 new signals falling at 5.54, 8.66, and 8.80 ppm could be observed. The peak at 5.54 ppm (marked with a full triangle in Figure 10) is assigned to the vinylic protons of DACHEX in the bis-adduct [Pt(5’-GMP)_2_(1,2-DACHEX)], while the signals at 8.66 and 8.80 ppm (marked with a full circle in Figure 10) are assigned to the H8 of the two coordinated 5’-GMPs, which are not equivalent due to the absence of a plane of symmetry passing through the cyclohexene ring. The three new signals grew in intensity with time, while the signals associated to complex **2** decreased.

The reaction of **1** and **2** with 5*’*-GMP was also performed at lower NaCl concentration (4 mM) in order to simulate the cytoplasmatic chloride concentration. The ^31^P NMR spectra recorded at pH* = 7.4 and 4 mM NaCl (see ESI, Supplementary Materials, Figures S1 and S2) appeared to be comparable to those obtained in the presence of 120 mM NaCl for both complexes **1** and **2**, indicating that the rate-determining step is the slow solvolysis of the pyrophosphate. These results are in line with the data already reported for **3** (no detectable binding to DNA after 7 days of incubation [20]) and **4** (slow reaction [24]).

### 2.6. Cytotoxicity Assays

**Figure 11:**
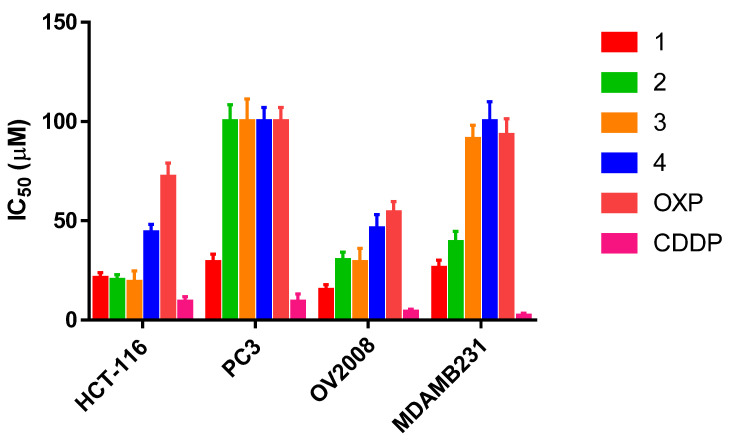
In vitro cytotoxicity of compounds **1**, **2**, **3**, **4**, oxaliplatin (OXP), and cisplatin (CDDP) towards HCT-116, PC3, OV2008, and MDAMB231 cell lines.

The in vitro cytotoxicity of the Pt-pyrophosphato complexes (**1**, **2**, **3**, and **4**) was evaluated on a panel of human cancer cell lines and compared to that of cisplatin and oxaliplatin. Cell lines representative of colon (HCT-116), prostate (PC3), breast (MDA-MB231), and cervical (OV2008) carcinomas were included. The cell viability was evaluated by means of the MTT test after 72 h of incubation with different concentrations of the tested compounds; the IC_50_ values, calculated from dose-survival curves, are plotted in Figure 11 and reported in Table S1, in the Supplementary Materials.

As expected, cisplatin was found to be the most effective in all cell lines (average IC_50_ = 6 μM) due to the faster substitution of the two chlorido ligands if compared to that of the bidentate pyrophosphato ligand. From this point of view, oxaliplatin represents a better reference compound, with the bidentate oxalato ligand being more similar to the bidentate pyrophosphato ligand. Compound **1** (average IC_50_ = 23 μM) was found to be much more effective than all other Pt-pyrophosphato complexes (**2**, **3**, and **4**) and oxaliplatin (average IC_50_ = 80 μM for OXP), and to possess a discrete activity also toward the prostate cancer cell line PC3 (IC_50_ = 29 µM, up to 3 times greater than that of the other complexes all of which have IC_50_ = 100 µM). Compound **2** follows, which is more active than **3**, **4**, and oxaliplatin in all cell lines except PC3. Finally, compound **3** was more active than **4** and oxaliplatin in HCT-116 and OV2008 cell lines (Figure 11).

### 2.7. Apoptosis Assay

**Figure 12:**
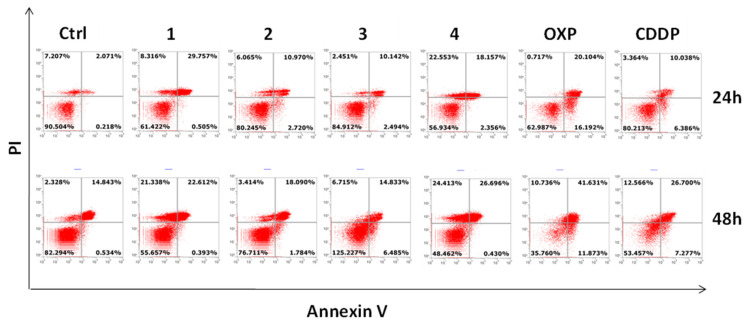
FCM dot plots of Annexin V/PI-positive HCT116 cells after 24 and 48 h incubation with compounds **1**–**4**, oxaliplatin (OXP), and cisplatin (CDDP) at their IC**_50_** concentrations (reported in Supplementary Table S1). The lower left quadrants of each panel show the viable cells. The upper left quadrants contain the necrotic cells. The upper right quadrants contain the late apoptotic cells. The lower right quadrants contain the early apoptotic cells. Each quadrant includes the corresponding % of cells measured by flow cytometry.

In order to obtain information on tumor cells’ death, the Annexin V/propidium iodide (PI) assay was used in flow cytometry analyses. Annexin V provides a very sensitive method for detecting cellular apoptosis, while PI is commonly used to detect necrotic or late apoptotic cells (when positive to Annexin too) [33]. The FITC Annexin V staining was carried out after 24 and 48 h of incubation of HCT116 cells with the Pt-pyrophosphato complexes (**1**–**4**) or oxaliplatin/cisplatin (used as reference compounds) at the IC_50_ concentrations (Supplementary Materials, Table S1). The Flow Cytometry (FCM) dot plots of Annexin V/PI-positive HCT116 cells are reported in Figure 12. The two-dimensional dot plots can be divided into four quadrants, corresponding to: (i) viable cells, which are negative to both probes (lower left quadrant; PI/FITC −/−), (ii) early apoptotic cells, which are PI-negative and Annexin V-positive (lower right quadrant; PI/FITC −/+), (iii) late apoptotic cells, which are Annexin- and PI-positive (upper right quadrant; PI/FITC +/+), and (iv) necrotic cells, which are PI-positive and Annexin-negative (upper left quadrant; PI/FITC +/−).

The data reported in Figure 12 show that, after 24 h incubation, apoptosis was more evident with **1** and **4** than with **3** and **2**. Moreover, only **1** and **4** induced necrosis. All compounds induced apoptosis to a lesser extent than oxaliplatin, but it must be taken into account that the IC**_50_** concentration of the latter drug (72 μM) was much higher than that of the other compounds (see Supplementary Materials, Table S1). After 48 h of incubation, the induction of apoptosis increased in all treated cells, while necrosis increased mainly in the case of **1** and **4**.

## 3. Materials and Methods

### 3.1. Reagents and Equipment

Commercial reagent-grade chemicals and solvents (Sigma, Milan, Italy) were used as received without further purification. ^1^H-, ^31^P-, and ^195^Pt-NMR and [^1^H-^13^C]-HSQC spectra were recorded on Bruker Avance DPX 300 MHz (Bruker Italia S.r.L., Milano, Italy) and Agilent Technologies 500 MHz (Agilent Technologies Italia S.p.A.; Cernusco sul Naviglio, Milano, Italy) instruments. Standard pulse sequences were used for ^1^H, ^31^P{^1^H}, and ^195^Pt{^1^H} (121.5, 242.9, and 64.5 MHz, respectively) one-dimensional spectra. ^1^H chemical shifts were referenced using the internal residual peak of the solvent (D_2_O: 4.80 ppm), ^31^P chemical shifts were referenced to external H_3_PO_4_ (85% *w*/*w*; 0 ppm), and ^195^Pt chemical shifts were referenced to external K_2_[PtCl_4_] in D_2_O fixed at −1628 ppm. Electrospray ionization mass spectrometry (ESI-MS) was performed with a dual electrospray interface and a quadrupole time-of-flight mass spectrometer (Agilent 6530 Series Accurate-Mass Quadrupole Time-of-Flight (Q-TOF) LC-MS) (Agilent Technologies Italia S.p.A.; Cernusco sul Naviglio, Milano, Italy). Elemental analyses were performed with an Eurovector EA 3000 CHN instrument (Eurovector S.p.A., Milano, Italy). A Crison Micro-pH meter Model 2002, equipped with Crison micro-combination electrodes (5 and 3 mm in diameter) (Hach Lange Spain, S.L.U.; Barcelona, Spain) and calibrated with Crison standard buffer solutions at pH 4.01, 7.02, and 10.00, were used for pH measurements. The pH readings from the pH-meter for D_2_O solutions are indicated as pH* values and are uncorrected for the effect of deuterium on glass electrodes [34]. HCT116 human colon cancer cells, PC3 human prostate cancer cells, and MDA MB 231 human breast cancer cells were purchased by ATCC**^®^**(ATCC, Manassas, VA, USA). OV2008 human cervical cancer cells were kindly gifted by Prof. G. J. Peters—Cancer Center Amsterdam. All necessary cell culture material was purchased from EuroClone (Pero, Milan, Italy). The MTT used for cytotoxicity studies was purchased from Sigma–Aldrich (Milan, Italy). The FITC Annexin V Apoptosis Detection Kit I was from BD PharmingenTM (San Jose, CA, USA, 556547).

### 3.2. Preparation and Characterization of Platinum Complexes

#### 3.2.1. [PtI_2_(*cis*-1,3-DACH)](DACH = diaminocyclohexane) 

K_2_PtCl_4_ (500 mg, 1.2 mmol) was dissolved in 20 mL of water and treated with 1.6 g of KI (8-fold excess). The reaction mixture was stirred for 5 min at room temperature and treated with 4 mL of a solution containing *cis*-1,3-DACH (0.145 mL, 1.21 mmol). A dark yellow precipitate formed immediately, and the resulting suspension was stirred at 40 °C for 2.5 h. The precipitate was isolated by filtration of the mother liquor, washed with cold water and diethyl ether, and dried under vacuum. Yield 86% (583 mg, 1.04 mmol). *Anal.* Calcd. for [PtI_2_(*cis*-1,3-DACH)] (PtI_2_N_2_C_6_H_14_): C, 12.79; H, 2.51; N, 4.97%. Found: C, 13.36; H, 2.61; N, 4.90%. ESI-MS calcd. for (C_6_H_14_N_2_I_2_PtNa) = 585.8743; found: *m/z* 585.8743 [M+Na]^+^. ^1^H-NMR (Acetone-d_6_): 3.25 (2H, CH_1/3_), 2.06 (1H, CH_2eq_), 4.82 (2H, NH_a_), 4.15 (2H, NH_b_), 4.65 (1H, CH_5ax_), 1.90 (1H, CH_5eq_), 1.77 (1H, CH_2ax_), and 1.71 (4H, CH_4/6_) ppm. See Figure 2 for numbering of *cis*-1,3-DACH protons.

#### 3.2.2. [Pt(CO_3_)(*cis*-1,3-DACH)]

A suspension of [PtI_2_(*cis*-1,3-DACH)] (244 mg, 0.433 mmol) in 20 mL of H_2_O was kept under stirring for 15 min and then Ag_2_CO_3_ (115.9 mg, 0.420 mmol) was added. After stirring the reaction mixture at 40 °C in the dark for 2 h, the suspension was filtered through a plug of Celite, to remove AgI, and the solvent was evaporated to dryness under reduced pressure to yield the desired compound [Pt(CO_3_)(*cis*-1,3-DACH)]. Obtained 106.4 mg (66.5% yield). ESI-MS calcd. for (C_7_H_14_N_2_O_3_PtNa) = 392.0501; found: *m/z* 392.0542 [M+Na]^+^.

#### 3.2.3. [Pt(CO_3_)(DMSO)_2_] (DMSO = dimethylsulfoxide)

The complex [Pt(CO_3_)(DMSO)_2_] was prepared according to an already reported procedure [35]. The characterization of the compound provided data in accordance with those reported in the literature.

#### 3.2.4. [PtI_2_(1*R*,2*R*-DACH)]

This complex was prepared following Dhara’s procedure [36].

#### 3.2.5. [Pt(CO_3_)(1*R*,2*R*-DACH)]

[PtI_2_(1*R*,2*R*-DACH)] (206.5 mg, 0.367 mmol) was suspended in 50 mL of H_2_O, kept under stirring for 15 min, and then treated with Ag_2_CO_3_ (98.1 mg, 0.356 mmol). After stirring the reaction mixture at 40 °C in the dark for 2 h, the suspension was filtered through a plug of Celite to remove AgI, and the solvent evaporated to dryness under reduced pressure to yield the desired compound [Pt(CO_3_)(1*R*,2*R*-DACH)]. Obtained 130.6 mg (96% yield). ESI-MS calcd. for (C_7_H_14_N_2_O_3_PtNa) = 392.0501; found: *m/z* 392.0538 [M+Na]^+^.

#### 3.2.6. Na_2_[Pt(Pyrophosphato)(*cis*-1,3-DACH)] (**1**; pyrophosphate = P_2_O_7_^4−^)

Tetrasodium pyrophosphate decahydrate (39.25 mg, 0.088 mmol) was dissolved in 5.0 mL of water at 55 °C, and the pH of the solution was adjusted to 8.0 with 1.0 M HClO_4_. After addition of [Pt(CO_3_)(*cis*-1,3-DACH)] (50 mg, 0.135 mmol) to the basic solution of pyrophosphate, the reaction mixture was first stirred for 3 h at 55 °C, then the pH was brought to 6.0 by addition of 1.0 M HClO_4_ and stirring resumed for a further 30 min at room temperature. The obtained suspension was filtered, and the solution was concentrated to a minimum volume (ca. 2 mL) by rotary evaporation. Addition of acetone induced the precipitation of the desired product as a white solid that was separated by filtration of the mother liquor and dried under vacuum. Obtained 35.5 mg (83% yield). *Anal.* Calcd. for Na_2_[Pt(pyrophosphato)(*cis*-1,3-DACH)]·2.5H_2_O (PtN_2_C_6_H_14_P_2_O_7_Na_2_·2.5H_2_O): C, 12.55; H, 3.33; N, 4.88%. Found: C, 12.95; H, 3.82; N, 4.36%. ESI-MS: calc. for [PtN_2_C_6_H_14_P_2_O_7_Na_2_]+Na^+^ = 551.9568; found: m/z = 551.9617 [M+Na]^+^. ^1^H-NMR (D_2_O): 2.73 (2H, CH_1/3_), 1.88 (1H, CH_2eq_), 1.56 (1H, CH_2ax_), 5.20 (4H, NH_a/b_), 4.53 (1H, CH_5ax_), 1.85 (1H, CH_5eq_), 1.74 (4H, CH_4/6_) ppm. ^31^P-NMR (D_2_O): 1.58 ppm. ^195^Pt-NMR (D_2_O): −1643 ppm. ^13^C{^1^H} NMR (D_2_O): 45.95 (C_1/3_), 36.13 (C_2_), 16.74(C_5_), 30.13 (C_4/6_) ppm. See Figure 2 for numbering of atoms.

#### 3.2.7. [Pt(Dihydrogenpyrophosphato)(1,2-DACHEX)] (**2**; 1,2-DACHEX = racemic *trans*-1,2-diamino-4-cyclohexene)

Tetrasodium pyrophosphate decahydrate (51.3 mg, 0.115 mmol) was dissolved in 10.0 mL of water at 55 °C, and the pH of the solution was adjusted to 8.0 with 1.0 M HNO_3_. The solution was kept under magnetic stirring at 55 °C for 15 min and then treated with [Pt(CO_3_)(DMSO)_2_] (50 mg, 0.121 mmol). After 5 min stirring, the reaction mixture was treated with a solution of *trans*-1,2-diamine-4-cyclohexene dihydrochloride (1.5 mL, 22.4 mg, 0.121 mmol), previously neutralized with KOH (1.0 M; 0.242 mmol), and stirred at 55 °C for a further 18 h, during which the pH was maintained at a constant value of ca. 8.0 by addition of aliquots of KOH (1.0 M), as required. After concentration of the reaction solution to half its initial volume (ca. 5 mL) by rotary evaporation, the pH was lowered to ca. 1–2 by addition of HNO_3_ (1.0 M), and acetone (100 mL) was added to induce the precipitation of the desired product as a white solid that was separated by filtration of the mother liquor and dried under vacuum. Obtained: 38.7 mg (70% yield). *Anal.* Calcd. for [Pt(pyrophosphato)(1,2-DACHEX)]·1.5H_2_O (PtN_2_C_6_H_14_P_2_O_7_·1.5H_2_O): C, 14.12; H, 3.35; N, 5.49%. Found: C, 14.46; H, 3.54; N, 5.03%. ESI-MS: calc. for [PtN_2_C_6_H_14_P_2_O_7_]-H= 481.9871; found: *m/z* = 481.9797 [M-H]^−^. ^1^H-NMR (D_2_O; pH*= 10 due to NaOD): 2.72 (2H, CH_c_), 5.45 (2H, CH_f_), 2.56 (2H, CH_e_), 2.16 (2H, CH_d_) ppm. ^31^P-NMR (D_2_O; pH*= 10): 1.79 ppm. ^195^Pt-NMR (D_2_O; pH*= 10): −1766 ppm. ^13^C{^1^H} NMR (D_2_O; pH*= 10): 127.76 (C_4/5_), 62.43 (C_1/2_), 35.43(C_3/6_) ppm. See Figure 4 for numbering of atoms.

#### 3.2.8. [Pt(Dihydrogenpyrophosphato)(1*R*,2*R*-DACH)] (**3**)

This complex was prepared by a modification of an already reported procedure [21]. Briefly, tetrasodium pyrophosphate decahydrate (39.25 mg, 0.088 mmol) was dissolved in 5.0 mL of water at 55 °C and the pH of the solution was adjusted to 8.0 with 1.0 M HNO_3_. The solution was kept under magnetic stirring at 55 °C for 15 min and then treated with [Pt(CO_3_)(1*R*,2*R*-DACH)] (50 mg, 0.135 mmol). The reaction mixture was stirred at 55 °C for 3.5 h and then concentrated to a minimum volume (ca. 2 mL) by rotary evaporation. After lowering the pH of the concentrated solution to ca. 1–2 by addition of HNO_3_ (1.0 M), acetone (100 mL) was added to induce the precipitation of the desired product as a yellow solid that was separated by filtration of the mother liquor and dried under vacuum. Obtained: 35.2 mg (82% yield). The characterization of **3** provided data in accordance with those reported in the literature [31].

#### 3.2.9. [Pt(Dihydrogenpyrophosphato)(*cis*-1,4-DACH)] (**4**)

This complex was prepared according to an already reported procedure and its characterization provided data in accordance with those reported in the literature [24].

### 3.3. NMR Experiments at Different pHs and Calculation of pKa Values

Acidity constants for complexes **1** and **2** were determined by ^31^P NMR chemical shift/pH titrations. Either compound **1** or **2** (0.0047 mmol) was dissolved in 0.550 mL of D_2_O and transferred into an NMR tube. The pH* of the sample was adjusted to the required value by addition of DClO_4_ (10, 1.0, 0.5, 0.1, or 0.05 M) or NaOD (1.0, 0.5, 0.1, or 0.05 M) solutions, and the pH* value was measured by using a 3 mm diameter electrode for NMR tubes. No control of the ionic strength was performed. The pH titration curves were fitted to the Henderson−Hasselbalch equation using the program KaleidaGraph (version 3.5, Synergy Software, Reading, PA, USA) [37].

### 3.4. Stability in Physiological or Acidic Conditions

The stability of compounds **1** and **2** at 37 °C in buffered solutions was assessed by ^31^P NMR spectroscopy. Each compound (~2 mg) was dissolved in 0.8 mL of D_2_O containing 50 mM 4-(2-hydroxyethyl)-1-piperazineethanesulfonic acid) (HEPES) buffer (pH* = 7.4 or pH* = 6.5) and 120 mM NaCl. The resulting solutions were transferred into NMR tubes and maintained at 37 °C. ^31^P NMR spectra were recorded from time to time over a period of one month.

### 3.5. Reactivity with 5′-GMP

Complex **1** (2 mg, 0.0041 mmol) or **2** (2 mg, 0.0041 mmol) was dissolved in 0.8 mL of D_2_O containing 50 mM HEPES buffer (pH* = 7.4) and 120 mM (to simulate the extracellular environment) or 4.0 mM (to simulate the intracellular environment) of NaCl. After addition of 4.1 mg (0.010 mmol) of guanosine 5′-monophosphate sodium salt (5′-GMP-Na·6H_2_O), the reaction mixture was transferred into an NMR tube that was maintained at 37 °C. The reactions were monitored up to one month by recording ^1^H and ^31^P NMR spectra at defined time intervals.

### 3.6. Cell Viability Assay

The number of living cells was evaluated by the 3-(4,5-dimethylthiazol-2-yl)-2,5-diphenyltetrazolium bromide (MTT) assay. Briefly, HCT116 human colon cancer cells, PC3 human prostate cancer cells, OV2008 human ovarian cancer cells, and MDA MB 231 human breast cancer cells were plated in 96-well plates at a density of ~5000 cells/well. After one day of incubation at 37 °C in a humid atmosphere with 5% CO_2_, the culture medium was replaced with 100 µL of fresh medium (control cells) or medium containing different concentrations of the test compounds in the range 1.56–100 µM. Untreated cells were used as positive controls. After the incubation period of 72 h, 10 µL of a 0.5% (*w*/*v*) MTT/PBS solution was added to each well and the incubation was prolonged for a further 2 h. After this period, the medium was removed and replaced with 100 µL of DMSO. The absorbance of each well was measured by a microplate reader (Wallac Victor3, 1420 Multilabel Counter, Perkin-Elmer, Singapore). Each compound concentration was tested in triplicate, and results were presented as percentage of the control value. IC_50_ values were calculated using non-linear regression in GraphPad Prism 5.01 (San Diego, CA, USA).

### 3.7. Annexin V/PI Apoptosis Assay

The Annexin V/PI assay was conducted as previously described [33] in order to investigate the mechanism of cell death induced by the various compounds on HCT116 cells. In particular, after 24 and 48 h of treatment with the compounds at concentrations equal to their IC_50_ values calculated by the MTT assay previously described, cells were subjected to Annexin-V FITC/propidium iodide staining, which allowed detection of early and late apoptosis as Annexin V-positive cells, late apoptosis as Annexin V/PI-positive cells, and necrotic cells as PI-positive cells. The FITC Annexin V Apoptosis Detection Kit I was from BD PharmingenTM (San Jose, CA, USA, 556547) and the analysis was performed on an Attune NxT acoustic focusing cytometer (Life Technologies, Thermo Fischer Scientific, Waltham, MA, USA). Data were interpreted using the CytExpert software v. 1.2 provided by the manufacturer.

## 4. Conclusions

Two new Pt(II) derivatives (**1** and **2**) with a pyrophosphato ligand, that differ from those already reported in the literature (**3** and **4**) for the diamine carrier ligand, have been synthesized and fully characterized with the aim of investigating how slight modifications in the carrier ligand can impact the antitumor activity of this class of drugs characterized by high bone tropism. Compounds **1** and **2** were synthesized, starting from the carbonate-precursor [Pt(CO_3_)(diamine)]. This procedure could also be applied to the synthesis of **3** with better results. ^31^P{^1^H} NMR experiments at different pH values have allowed the determination of the acidity constants of complexes **1** and **2** and an estimation of their stability in physiological solutions. The complexes are more stable in physiological conditions than in acidic conditions (characteristic of the tumor sites) and compound **1** appeared to be slightly less reactive than compound **2**.

NMR studies on the reactivity of **1** and **2** towards 5’-GMP (used as a model of nucleic acid) showed that both compounds react slowly with 5’-GMP, forming the bis-adduct, and that the rate does not appear to depend upon the NaCl concentration (120 mM to mimic the extracellular chloride concentration and 4 mM to mimic the intracellular concentration). Finally, in a preliminary pharmacological investigation, the in vitro cytotoxicity assays, performed against a panel of four human cancer cell lines, showed an activity order of **1** > **2** > **3** > **4** > oxaliplatin. Cisplatin, that resulted to be the most active of all compounds, cannot be taken as a reference since it contains monodentate chlorido ligands which are known to be much more hydrolysable than the bidentate pyrophosphate and oxalate. Interestingly, the *cis*-1,3-DACH diamine ligand appears to confer the best cytotoxic effect. Flow cytometry studies performed to investigate the mechanism of cell death induced by compounds on HCT116 cells showed that the Pt(II)-pyrophosphato compounds with the non-classical 1,3- and 1,4-diaminocyclohexane ligands (**1** and **4**) are the most capable to induce cell death by apoptosis. A future perspective will be a deeper pharmacological investigation of the new Pt(II)-pyrophosphato derivatives, focusing on their potential selectivity towards bone tumors as well as their embodiment in hydroxyapatite matrices to be used for the local treatment of bone tumors after implantation.

## Data Availability

The data presented in this study are available upon request from the corresponding author.

## Supplementary Materials

The following are available online. Scheme S1: synthesis of compound **2**. Scheme S2: synthesis of compound **3**. Figure S1: ^31^P NMR spectra at different time intervals of **1** in the presence of 5’-GMP. Figure S2: ^31^P NMR and ^1^H NMR spectra at different time intervals of **2** in the presence of 5’-GMP. Table S1: in vitro cytotoxicity data (IC_50_).

## Sample Availability

Samples of the compounds **1**–**4** are available from the authors.
