# Supplementary material for: New Oxaliplatin-Pyrophosphato Analogs with Improved In Vitro Cytotoxicity
Source: Molecules. 2021 Jun 4;26(11):3417. doi: 10.3390/molecules26113417 (PMC8200237; doi:10.3390/molecules26113417)
Supplement: Supplementary file 1 [file molecules-26-03417-s001.zip › molecules-1224562-supplementary.pdf]

ELECTRONIC SUPPLEMENTARY MATERIALS

New Oxaliplatin-Pyrophosphato Analogs with Improved In Vitro Cytotoxicity.

Alessandra Barbanente,[1] Rosa Maria Iacobazzi,[2] Amalia Azzariti,[2] James D. Hoeschele,[3] Nunzio Denora,[4] Paride Papadia,[5] Concetta Pacifico,[1] Giovanni Natile,[1] Nicola Margiotta.[1,*]

[1]Dipartimento di Chimica, Università degli Studi di Bari Aldo Moro, Via E. Orabona 4, 70125 Bari, Italy.

[2]Experimental Pharmacology Laboratory, IRCCS Istituto Tumori Giovanni Paolo II, Bari, Italy.

[3]Department of Chemistry, Eastern Michigan University, 48197 Ypsilanti, Michigan, United States.

[4]Dipartimento di Farmacia-Scienze del Farmaco, Università degli Studi di Bari Aldo Moro, Via E. Orabona 4, 70125 Bari, Italy;

[5]Department of Biological and Environmental Sciences and Technologies (DiSTeBA), University of Salento, Prov.le Lecce-Monteroni, Centro Ecotekne, 73100 Lecce, Italy.

*Correspondence: nicola.margiotta@uniba.it; Tel.: +39 080 5442759

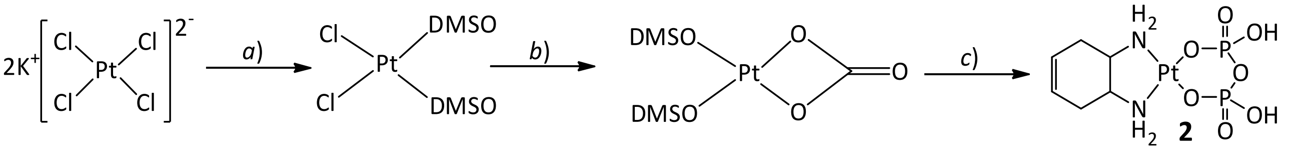

Scheme S1. *a)* water, dimethyl sulfoxide, room temperature, dark, 24 h; *b)* water, Ag_2CO_3, 40 °C, 2 h; *c)* water, pH=8, *trans*-1,2-diamino-cyclohexene (DACHEX), sodium pyrophosphate decahydrate, 55 °C, 18 h.

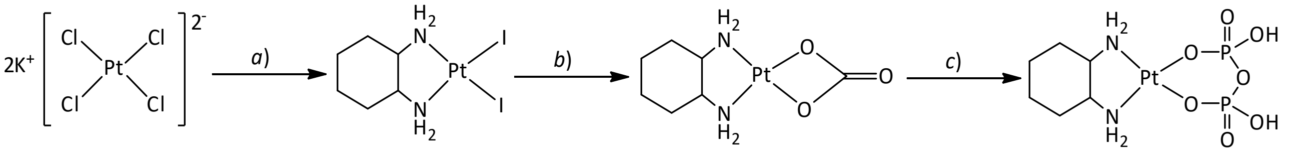

Scheme S2. *a)* water, excess KI (6 fold), 1*R*,2*R*-diamino-cyclohexane (DACH), room temperature, 3 h; *b)* water, Ag_2CO_3, 40 °C, 2 h; *c)* water, pH=8, sodium pyrophosphate decahydrate, 55 °C, 3.5 h.

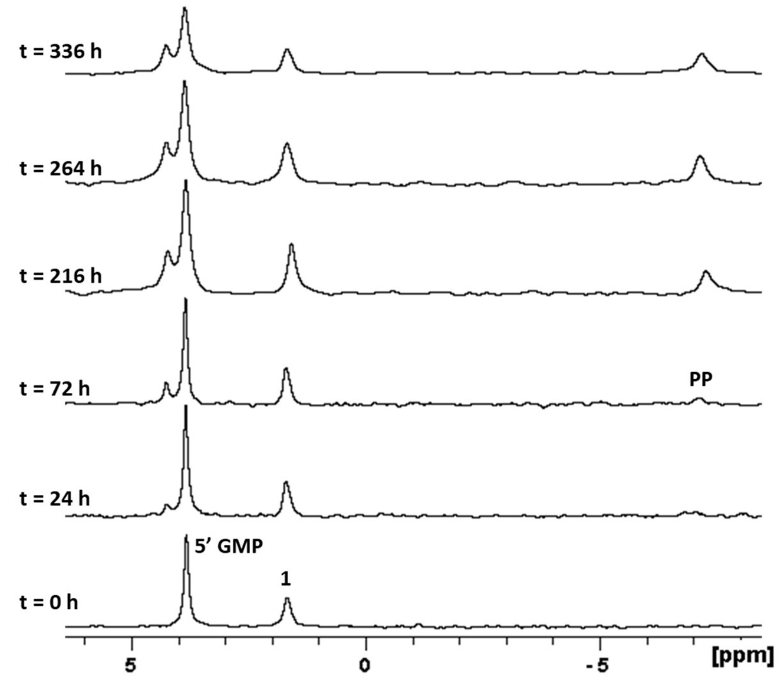

Figure S1. ^{31}P NMR spectra at different time intervals of **1** (5 mM) in the presence of 5'-GMP (12.5 mM) at pH* = 7.4 (D_2O, 50 mM HEPES buffer), 4 mM NaCl, and 37 °C.

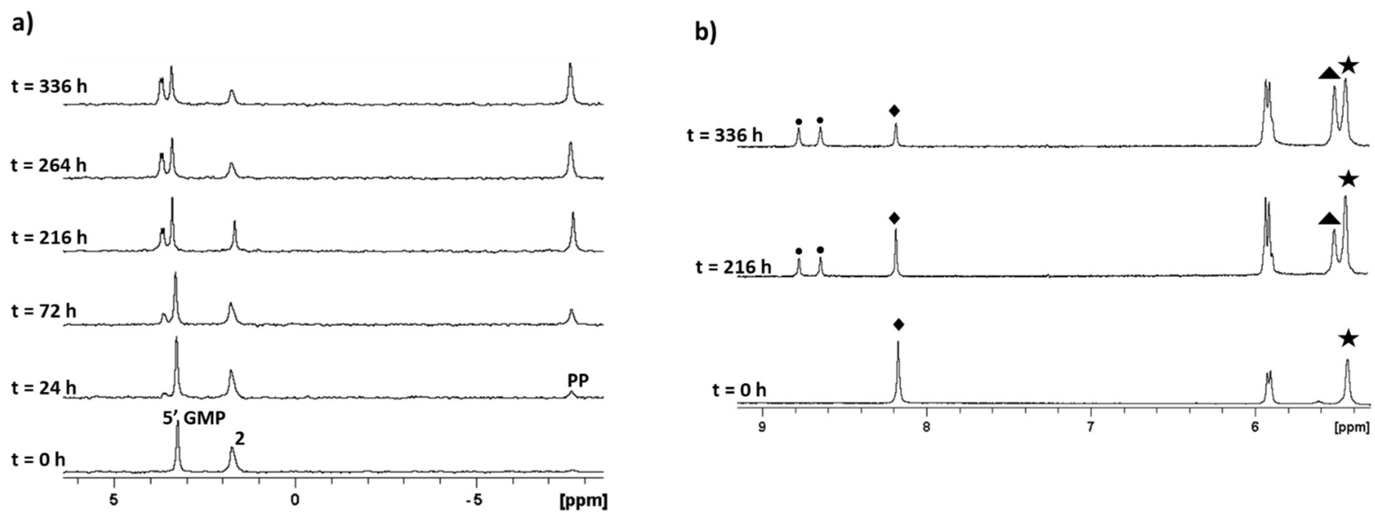

Figure S2. a) ^{31}P NMR spectra at different time intervals of **2** (5 mM) in the presence of 5'-GMP (12.5 mM) at pH* = 7.4 (D_2O, 50 mM HEPES buffer), 4 mM NaCl, and 37 °C. b) ^{1}H NMR spectra at different time intervals of **2** (5 mM) in the presence of 5'-GMP (12.5 mM) at pH* = 7.4 (D_2O, 50 mM HEPES buffer), 4 mM NaCl, and 37 °C.

Table S1. In *vitro* cytotoxicity[a]

Compounds	IC$_{50}$ (µM) ± S.D.				
	**HCT116**	**PC3**	**OV2008**	**MDAMB231**	***Average***
**1**	21±2	29±3	15±2	26±3	23±3
**2**	20±2	100±6	30±3	39±4	47±6
**3**	19±4	100±8	29±5	91±5	60±8
**4**	44±3	100±5	46±5	100±7	72±7
**CDDP**	9±2	9±3	4±1	2±1	6±3
**OXP**	72±5	100±5	54±4	93±6	80±6

[a]Cells (3–5·10^{3} cells per mL) were treated for 72 h with increasing concentrations of the tested compounds. Cytotoxicity was assessed by the MTT test. IC$_{50}$ values were calculated by the four parameter logistic model ($p < 0.05$). S.D. = standard deviation. OXP = oxaliplatin, CDDP = cisplatin.
